# Clinical and Pathological Characteristics of Organized Hematoma

**DOI:** 10.1155/2013/539642

**Published:** 2013-03-06

**Authors:** Nobuo Ohta, Tomoo Watanabe, Tsukasa Ito, Toshinori Kubota, Yusuke Suzuki, Akihiro Ishida, Masaru Aoyagi, Atsushi Matsubara, Kenji Izuhara, Seiji Kakehata

**Affiliations:** ^1^Department of Otolaryngology, Head and Neck Surgery, Faculty of Medicine, Yamagata University, 2-2-2 Iida-nishi, Yamagata 990-9585, Japan; ^2^Department of Otorhinolaryngology, Hirosaki University Graduate School of Medicine, Hirosaki 036-8562, Japan; ^3^Division of Medical Biochemistry, Department of Biomolecular Sciences, Faculty of Medicine, Saga University, Saga 840-8502, Japan

## Abstract

*Objective*. To study the clinical and pathological characteristics of patients with organized hematoma with malignant features in maxillary sinuses. *Subjects and Methods*. This was a retrospective study of five patients who were treated surgically for organized hematoma. The preoperative CT and MRI findings were studied clinically. The expressions of CD31, CD34, and periostin in surgical samples were investigated by immunohistochemistry. *Results*. The clinical features of organized hematoma, such as a mass expanding from the maxillary sinus with bone destruction, resembled those of maxillary carcinoma. However, CT and MRI provided sufficient and useful information to differentiate this condition from malignancy. Surgical resection was the first-line treatment because of the presence of a firm capsule. Characteristic histopathological findings were a mixture of dilated vessels, hemorrhage, fibrin exudation, fibrosis, hyalinization, and neovascularization. The expressions of periostin, CD31, and CD34 were observed in organized hematoma of the maxillary sinus. *Conclusion*. The expressions of periostin, CD31, and CD34 were observed in organized hematoma of the maxillary sinus. Organized hematoma is characterized pathologically by a mixture of bleeding, dilated vessels, hemorrhage, fibrin exudation, fibrosis, hyalinization, and neovascularization. CT and MRI show heterogeneous findings reflecting a mixture of these pathological entities.

## 1. Introduction

Nonneoplastic hemorrhagic lesions causing mucosal swelling and bone destruction can develop in the maxillary sinus. This type of lesion was reported in the Japanese literature in 1917 as a “blood boil of the maxillary sinus” by Tadokoro and is comparatively well known in Japan as one of the differential diagnoses of maxillary carcinoma. However, in the English literature, this type of lesion tends to be referred to as hemangioma of the maxillary sinus, organized hematoma of the maxillary sinus, or organized hematoma of the maxillary sinus mimicking tumor [1–8]. Although their clinical manifestations are very similar, the relationships between these entities have not been described.

We recently resected five such lesions and examined the associated clinical and histological features. Histologically, a combination of dilated vessels, hemorrhage, fibrin exudation, fibrosis, hyalinization, and neovascularization was characteristic. The lesion mimicked not only hematoma but also hemangioma. Therefore, either of the terms hemangioma or hematoma reflects the complete histological picture. In this paper, we report the clinicopathological characteristics of this entity and the relationship between the imaging and histopathological findings.

## 2. Subjects and Methods

### 2.1. Subjects

To evaluate the clinicopathological entity of the organized hematoma, we recruited subjects with lesions that met the following criteria. (1) CT demonstrated an expanding unilateral maxillary lesion, with thinning or destruction of the surrounding bony tissue; (2) MRI demonstrated a heterogeneous mass; (3) macroscopically, a mass with a hemorrhagic and heterogeneous appearance was observed; (4) histologically, nonneoplastic tissue with mucosal hemorrhage was observed.

Of all the patients referred to our department between 1996 and 2010 with suspected maxillary tumor, five met these criteria. These patients underwent clinical evaluation, followed by either transmaxillary or endonasal endoscopic sinus surgery and histopathological examination of the resected tissue.

### 2.2. Immunohistochemistry to Detect CD31, CD34, and Periostin

For immunohistochemical detection of CD31, CD34, and periostin, we used the labeled streptavidin-biotin-complex (SABC) method. Deparaffinized tissue sections were rehydrated in alcohols. The sections were autoclaved for 10 min at 120°C in citrate phosphate buffer (pH 6.0) for antigen retrieval. Endogenous peroxidase activity was blocked with 0.3% H_2_O_2_ for 30 min. The sections were then incubated with skim milk normal in phosphate-buffered saline (PBS) for 10 min to block nonspecific background staining. Monoclonal anti-CD31 and CD34 antibodies were purchased from Dako Japan (Tokyo, Japan). A polyclonal anti-Pendrin or anti-Periostin antibody was generated by immunising the rabbits with specific peptides. Polyclonal antibody against Pendrin was applied as a primary antibody at a dilution of 1 : 100 and incubated at 4°C overnight. Polyclonal antibody against Periostin was applied as a primary antibody at a dilution of 1 : 500 and incubated at 4°C overnight. After the sections had been washed with PBS, biotinylated goat anti-rabbit IgG was applied, and they were then incubated for 1 h at room temperature. Slides were developed by using diaminobenzidine and were counterstained with hematoxylin.

### 2.3. Assessment of Slides

Immunostained sections were assessed at 200x  magnification under an Olympus microscope with an eyepiece reticle. Cell counts are expressed as means per high-power field (0.202 mm^2^). At least two sections were immunostained, and more than five areas were evaluated via the reticle. Results are expressed as number of positive cells per field, as follows: (−): negative; (+): fewer than 10 cells in each high-power field (×400); (++): 10 to 20 cells; (+++): more than 20 cells.

## 3. Results

**Figure 1:**
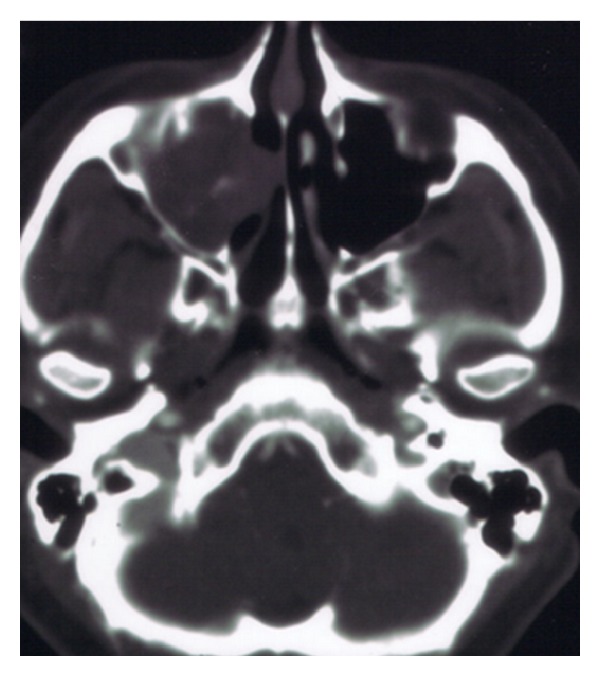
A 36-year-old female had a 2-month history of recurrent epistaxis. Initial coronal contrast-enhanced computed tomography scan of the paranasal sinuses revealed a 36 × 24 mm heterogeneous enhanced mass in the right maxillary sinus. The central region of the mass was strongly enhanced. Compression and thinning of the lateral wall of the right nasal cavity were observed (case III).

**Figure 2:**
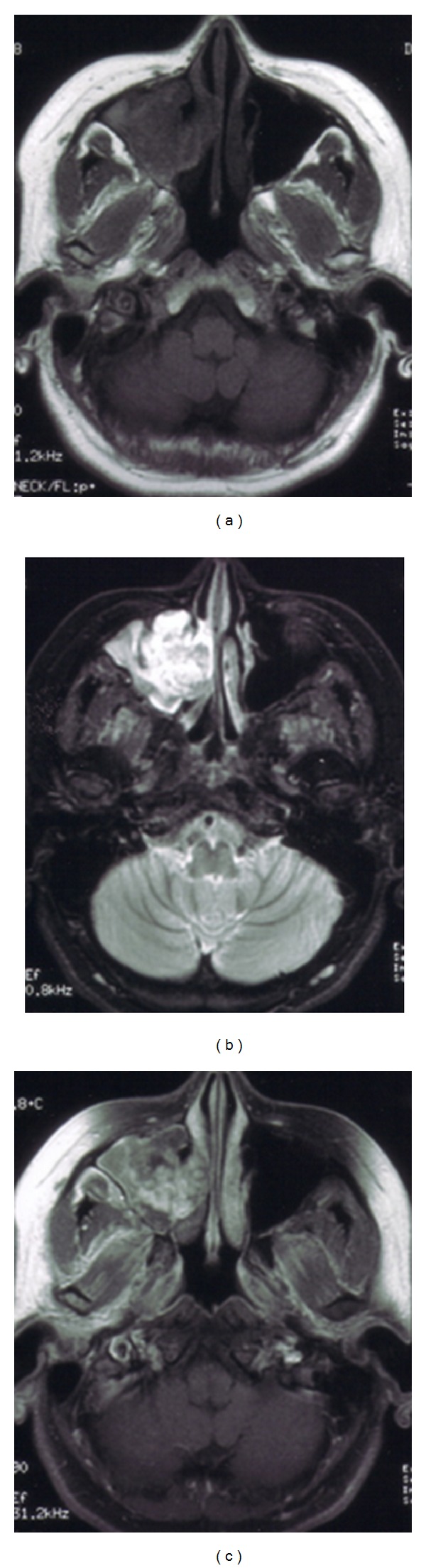
A 36-year-old female had a 2-month history of recurrent epistaxis. (a) Initial T1-weighted magnetic resonance image before treatment, showing a slightly low intensity in the central region of the mass (coronal view). (b) Initial T2-weighted magnetic resonance image before treatment, showing a heterogeneous mass. The central portion had high signal intensity, and the surrounding region had lower signal intensity than the central portion. The paranasal mucosa was thickened but kept its structure (axial view). (c) Initial Gd-DTPA magnetic resonance image before treatment, showing that the central portion was strongly enhanced. The surrounding region was less enhanced than the central portion. The thickened mucosa of the paranasal sinus was also strongly enhanced (axial view).

Patient characteristics and the clinical features of the five cases are summarized in Table 1. The age of the patients ranged from 14 to 56 years. The clinical features resembled those of maxillary carcinoma. The most frequently observed primary sign was recurrent epistaxis, although a wide variety of clinical features were observed, including nasal obstruction, cheek pain, and nasal pain. In most cases, the clinical course before diagnosis had been a for few months; however, one patient visited an ENT clinic because of hemorrhage from right nasal cavity mass and right nasal obstruction that had gradually enlarged over the course of 11 months. No patient exhibited coagulation abnormalities or reported a history of facial trauma. CT showed unilateral maxillary masses with thinning or destruction of the surrounding bony structures (Figure 1). The expansion pattern was not invasive. On MRI, the masses were well demarcated from the surrounding structures and heterogeneous in signal density on both T1-weighted, T2-weighted, and Gd-DTPA-enhanced images (Figure 2). CT and MRI provided sufficient information to differentiate these lesions from malignancy (Table 2).

A transmaxillary surgical approach was used in two patients and endonasal endoscopic sinus surgery in three patients. None of the patients required a blood transfusion as a result of intraoperative bleeding. The masses had firm capsules and were therefore removed easily, even in all cases in which the lesion extended from the maxillary sinus. Before the surgery, three patients underwent angiography, which revealed that the maxillary artery was the feeder artery. Two of the patients had strongly enhanced lesions, which were embolized with Gelform (Table 3).

**Figure 3:**
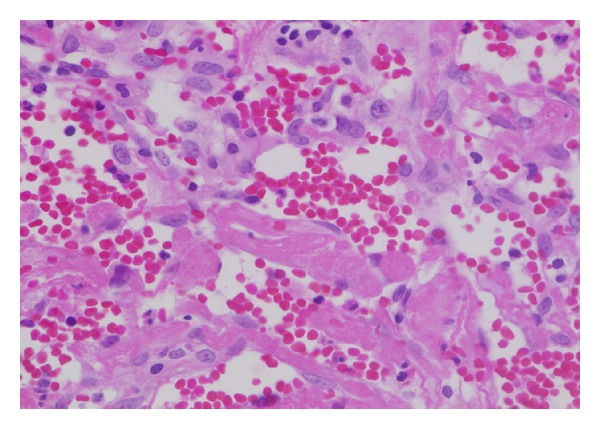
Representative pathological findings of organized hematoma. Nonneoplastic tissue with hemorrhage, fibrin exudation, and hyalinization was observed. (HE, original magnification ×100).

**Figure 4:**
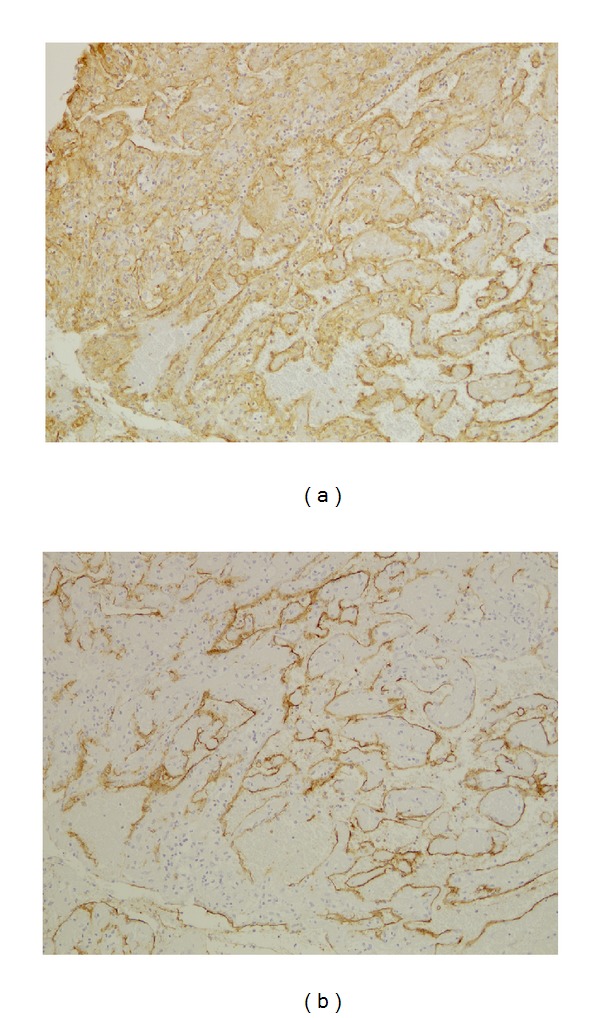
Immunohistochemical staining for CD31 and CD34 in organized hematoma (original magnification ×100). CD31-positive cells were observed in the organized hematoma, and dilated vessels were found (a). CD34-positive cells were observed in the organized hematoma (b). (Immunostaining, original magnification ×100 and ×200.)

**Figure 5:**
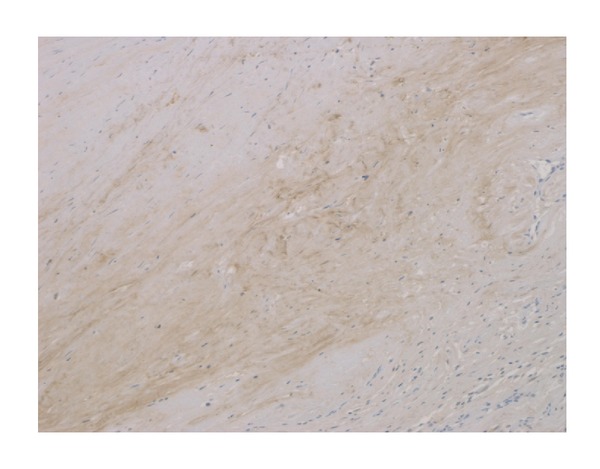
Immunohistochemical staining for periostin in organized hematoma (original magnification ×100). The expression of periostin was observed in the organized hematoma.

Histologically, the specimens consisted of dilated vessels, hemorrhage, fibrin exudation, fibrosis, hyalinization, and neovascularization, without tumor cells (Figure 3). CD31-positive cells (endothelial cells) were identified in separate sections from each mass. CD34-positive cells (progenitors of endothelial cells) were also observed in the specimens (Figure 4). The expression of periostin was also observed in the specimens (Figure 5). These characteristic findings were generally seen in separate sections from each mass; the pattern of the mixture of findings differed among parts of the same mass, masses from the same patient, and patients. The appearance of the findings also varied among different parts of the masses and among patients. To obtain all of these findings, we needed to examine several sections from different parts of each mass (Table 4). These characteristic findings were all subepithelial, and the lining of the epithelium was intact.

Followup to date (range from 6 to 108 months) has demonstrated all patients to be free of recurrence.

## 4. Discussion

Preoperative CT showed a heterogeneously enhanced mass expanding out of the maxillary sinus, with thinning or destruction of the adjacent bone. This expansion did not exhibit an invasive pattern but is commonly seen in malignancy, so the CT findings were not specific to this disease. This limitation was overcome by using MRI. MRI was able to demonstrate the apparent morphological heterogeneity of this disease and thus clearly differentiated it from neoplasms and mucoceles. A heterogeneous signal intensity on MRI was commonly observed. MRI showed thickening of the paranasal sinus mucosa surrounding the mass. The mucosa was well enhanced on T1-weighted images with contrast and had high signal intensity on T2-weighted images. These findings suggested the presence of inflammatory change due to obstruction by the lesion. In addition, the central part of the lesion had low signal intensity on T1-weighted imaging and high signal intensity on T2-weighted imaging and was well enhanced. These findings suggested that this central region contained blood with a low flow speed, thus matching the pathological finding of hematoma. The periphery was less well enhanced, thus matching the zone of fibrosis. This biphasic appearance is an important imaging aspect of this lesion and needs to be considered in the differential diagnosis before a decision is made on a treatment strategy. Recognizing the location of the hematoma preoperatively could be useful for avoiding intraoperative bleeding from the mass.

Microscopic examination revealed dilated vessels, hemorrhage, fibrin exudation, fibrosis, hyalinization, and neovascularization in the subepithelium. The appearance of these findings varied among different parts of the masses, and as for the disease etiology, Omura et al. suggest the negative spiral theory during the healing process as follows [8]. First, a blood clot accumulates in the closed space owing to bleeding of various causes, including hemangioma formation, facial injury, or inflammation. Next, necrosis, fibrosis, and hyalinization occur in turn, and neovascularization develops as part of the biological healing processes. As a result, the new vessels become weak, and rebleeding might easily occur. To examine this negative spiral theory immunohistopathologically, CD31, CD34, and periostin were used for immunostaining. These results showed that the endothelial cells kept their structural integrity and dilated pattern in limited areas. CD 34 is expressed on the progenitors of endothelial cells and might play an important role in neovascularization and wound healing [9–11]. CD34 immunostaining showed that these endothelial cell progenitors were present in the lesions. Periostin is a regulator of fibrosis and collagen deposits, and although it has been recognized for the important role it plays in myocardial repair/remodeling following myocardial infarction [12], there are indicators that its overexpression in the nasal mucosa contributes to tissue repair and fibrosis. These findings may support the negative spiral theory immunohistopathologically.

Surgery is mandatory to remove the organized hematoma as first-line treatment. To differentiate the organized hematoma from a malignant tumor, the complete absence of neoplastic cells in the totally removed specimen should be confirmed [1–8]. Thus, total removal under general anesthesia is usually performed, and the surgical procedure should be chosen depending on the size of the lesion. ESS should be considered as the first line in limited lesion with adequate working space for the endoscopic operative field. In case of large lesion with thinning the bone structure of the paranasal sinuses, the transmaxillary approach should be chosen. There was little difference in the intraoperative bleeding volume between patients who received endoscopic sinus surgery (ESS) and those who underwent the transmaxillary approach. To decrease the intraoperative bleeding volume, embolization of the feeder artery is necessary if the feeder artery is identified by angiography. In our series, angiography was performed in only three patients and embolization in two. In the two patients that received embolization, the volume of intraoperative bleeding was less than that in the patients who did not receive embolization.

In summary, we have reported the distinct clinicopathological entity of organized hematoma. Organized hematoma of the maxillary sinus was characterized pathologically by a mixture of bleeding, dilated vessels, hemorrhage, fibrin exudation, fibrosis, hyalinization, and neovascularization. CT and MRI gave heterogeneous findings reflecting a mixture of these pathological conditions.

## Figures and Tables

**Table 1:** Patient demographics and clinical features.

Case	age/gender	Chief complaint	Location	Side	Intranasal findings	Duration (m)
I	38/M	Epistaxis	MS	R	Mass in the MM	1
II	14/M	Epistaxis	MS	L	Mass in the MM	3
III	36/F	Nasal obstruction	MS	R	Polyps in the MM	2
IV	31/M	Nasal obstruction	MS	R	Bulging of the LW	6
V	56/F	Epistaxis	MS	R	Mass in the MM	11

M: male; F: female; MS: maxillary sinus; R: right; L: left; MM: middle meatus; LW: lateral wall.

**Table 2:** The CT and MRI findings of the five cases.

Case	Size on CT (mm)	CT (bone destruction)	MRI (T1-weighted)		MRI (T2-weighted)		MRI (Gd-DPTA)	
			Central	Surrounding	Central	Surrounding	Central	Surrounding
I	43 × 57 × 46	+(L,P)	Low	Low	High	Iso to high	High	Low
II	37 × 28 × 30	+(L)	Low	Low to iso	High	Iso to high	High	Low
III	30 × 31 × 28	+(L)	Low	Low	High	high	Iso to high	Low to iso
IV	35 × 31 × 29	+(L)	Low	Low to iso	High	high	Iso to high	Low to iso
V	25 × 30 × 27	+(L,P)	Low	Low to iso	High	Iso to high	High	Low

L: lateral wall of nasal cavity; P: posterior wall of maxillary sinus; Low: low signal intensity; iso: iso signal intensity; high: high signal intensity; central: central portion of the mass; surrounding: surrounding portion of the mass; Gd-DPTA: Gd-DPTA enhanced.

**Table 3:** Treatment and outcomes.

Case	Angiography	Feeding artery	Embolization	Operation method	Bleeding volume (mL)	followup (m)	recurrence
I	(+)	(−)	(−)	Transmaxillary	56	108	none
II	(+)	MA	Gelform	ESS	5	48	none
III	(+)	MA	Gelform	ESS	8	6	none
IV	(−)			ESS	65	12	none
V	(−)			Transmaxillary	120	76	none

(+): performed; (−): not performed or not confirmed; MA: maxillary artery; m: months; ESS: endonasal endoscopic sinus surgery; transmaxillary: transmaxillary approach with sublabial incision.

**Table 4:** Pathological findings.

Case	Bleeding	Necrosis	Fibrosis	Hyalinization	neovascularization	Vascular dilatation	CD31	CD34
I	(+)	(+)	(+)	(++)	(++)	(+)	(+++)	(++)
II	(+)	(+)	(++)	(++)	(+)	(+)	(++)	(+)
III	(+)	(−)	(+)	(+)	(+)	(−)	(+)	(+)
IV	(+)	(−)	(+)	(+)	(+)	(−)	(+)	(+)
V	(+)	(++)	(+)	(+)	(++)	(+)	(++)	(++)
